# Redox Homeostasis as a Therapeutic Target in Chronic Oxidative Diseases: Implications for Cancer Treatment

**DOI:** 10.3390/antiox15020203

**Published:** 2026-02-03

**Authors:** Moon Nyeo Park, Min Choi, Rony Abdi Syahputra, Domenico V. Delfino, Seong-Gyu Ko, Bonglee Kim

**Affiliations:** 1 College of Korean Medicine, Kyung Hee University, 1-5 Hoegidong, Dongdaemun-gu, Seoul 02447, Republic of Korea; mnpark@khu.ac.kr (M.N.P.); chlals2078@khu.ac.kr (M.C.); 2 Department of Pharmacology, Faculty of Pharmacy, Universitas Sumatera Utara, Medan 20155, Sumatera Utara, Indonesia; rony@usu.ac.id; 3 Department of Medicine and Surgery, University of Perugia, Piazza Università 1, 06123 Perugia, Italy; domenico.delfino@unipg.it; 4 Korean Medicine-Based Drug Repositioning Cancer Research Center, College of Korean Medicine, Kyung Hee University, Hoegi-dong Dongdaemun-gu, Seoul 02447, Republic of Korea

**Keywords:** redox signaling, redox homeostasis, chronic oxidative diseases, redox reprogramming, redox biomarkers, precision redox therapy, cancer redox adaptation, metabolic and neurodegenerative disorders, natural redox modulators, clinical redox medicine

## Abstract

Reactive oxygen species (ROS) have traditionally been viewed as pathological by-products of metabolism that drive tissue damage through oxidative stress. However, accumulating evidence across chronic diseases, including metabolic, cardiovascular, neurodegenerative disorders, and cancer, indicates that ROS also function as tightly regulated signaling molecules essential for cellular adaptation and survival. This paradigm shift from oxidative stress to redox signaling necessitates a fundamental re-evaluation of how redox imbalance contributes to chronic disease pathogenesis. In this review, we propose that chronic diseases should be understood as disorders of maladaptive redox homeostasis rather than simple consequences of excessive oxidative damage. We delineate the distinction between oxidative stress and redox signaling, emphasizing how chronic redox remodeling stabilizes pathological cellular states through coordinated regulation of key redox-sensitive transcriptional nodes, including KEAP1–NRF2, FOXO, HIFs, and NF-κB. Using cancer as a representative model, we illustrate how elevated but buffered ROS levels support oncogenic signaling, metabolic rewiring, and therapeutic resistance through redox addiction. We further discuss why non-specific antioxidant strategies have largely failed and argue that effective intervention requires context-dependent redox modulation rather than global ROS suppression. Finally, we introduce therapeutic redox reprogramming and outline future directions for precision redox medicine based on biomarker-guided stratification and disease stage-specific targeting strategies.

## 1. Introduction

### Redox Signaling vs. Oxidative Stress

Reactive oxygen species (ROS) have long been regarded primarily as harmful by-products of aerobic metabolism that induce cellular damage through lipid peroxidation, protein oxidation, and genomic instability. This damage-centric view formed the conceptual foundation of oxidative stress, a pathological state in which excessive ROS overwhelm endogenous antioxidant defenses and cause irreversible molecular injury [1]. However, accumulating biochemical and physiological evidence has established that ROS also function as tightly regulated second messengers that participate in signal transduction, adaptation, and cell fate determination—a paradigm now defined as redox signaling [2,3]. At the mechanistic level, the distinction between oxidative stress and redox signaling is not qualitative but quantitative and kinetic. Redox signaling is mediated predominantly by low, spatially confined concentrations of hydrogen peroxide (H_2_O_2_) that selectively oxidize reactive cysteine residues within target proteins, forming reversible sulfenic (–SOH) modifications [2,4]. These reversible oxidative events induce conformational changes that regulate kinase activity, phosphatase inhibition, transcription factor activation, and metabolic enzyme flux, thereby functioning analogously to phosphorylation-based signaling [3]. In contrast, oxidative stress arises when ROS levels exceed buffering capacity, leading to irreversible oxidation of cysteine residues to sulfinic (–SO_2_H) or sulfonic (–SO_3_H) states, as well as indiscriminate damage to lipids, DNA, and proteins [1,4].

This redox signaling–oxidative stress dichotomy explains why ROS exhibit hormetic behavior across biological systems. Low-level ROS pulses are essential for physiological processes including mitochondrial biogenesis, immune activation, stem cell differentiation, and adaptive stress responses, whereas sustained or excessive ROS accumulation drives pathology [3,5]. Importantly, cells possess highly specialized enzymatic systems—such as peroxiredoxins, glutathione peroxidases, and the thioredoxin system—that actively shape ROS gradients, enabling signaling while preventing toxic diffusion [2,6]. Thus, redox homeostasis reflects a regulated signaling architecture rather than passive detoxification. Failure to distinguish redox signaling from oxidative stress has contributed to the limited clinical efficacy of antioxidant therapies, as large-scale trials using non-specific agents such as vitamin E and ascorbic acid were based on the flawed assumption that global ROS neutralization is therapeutically beneficial [7]. However, these interventions often failed or even worsened outcomes, in part because indiscriminate ROS scavenging disrupts essential redox signaling required for cellular adaptation and immune competence [7,8]. Moreover, the extremely short half-life of free radicals compared to administered antioxidants renders stoichiometric scavenging biologically implausible in vivo, particularly in chronic diseases characterized by long-term redox remodeling rather than acute oxidative injury [8].

In cancer and other chronic diseases, redox signaling becomes progressively rewired rather than simply elevated. Tumor cells, for example, often operate within redox-adapted states in which ROS production is sustained but precisely buffered, enabling oncogenic signaling, metabolic plasticity, and resistance to therapy [9]. In such contexts, oxidative stress markers may be elevated, yet the dominant biological output is controlled redox signaling rather than lethal damage. This distinction is critical, as therapeutic strategies aimed at suppressing oxidative stress may inadvertently stabilize redox-adapted disease states rather than disrupt them. Collectively, these observations establish that oxidative stress and redox signaling represent fundamentally distinct biological regimes. Oxidative stress reflects a failure of control and accumulation of damage, whereas redox signaling constitutes an evolutionarily conserved communication system that governs cellular adaptation. Therapeutic success in chronic oxidative diseases—including cancer—therefore depends not on blanket antioxidant suppression but on precise modulation of redox signaling nodes that determine cell survival, plasticity, and stress tolerance [3,6,9].

## 2. Redox Homeostasis in Chronic Disease

### 2.1. Chronic Disease as Disorders of Maladaptive Redox Homeostasis

Chronic diseases, including cardiovascular disorders, metabolic syndromes, neurodegenerative diseases, and cancer, share a common pathological feature: a persistent disruption of redox homeostasis that differs fundamentally from acute oxidative stress. Rather than transient ROS overload, these conditions are characterized by long-term reprogramming of redox signaling pathways that stabilize disease-associated cellular states [10,11]. This maladaptive redox remodeling enables cells and tissues to survive under continuous metabolic, inflammatory, or environmental stress while progressively losing physiological flexibility. In cardiovascular diseases, sustained activation of redox-sensitive signaling pathways—particularly those involving NADPH oxidases, mitochondrial ROS, and inflammatory transcription factors—drives endothelial dysfunction and vascular remodeling without inducing overt cell death [12,13]. These observations indicate that pathological redox signaling can persist at sublethal levels, continuously reshaping cellular behavior while avoiding catastrophic oxidative damage. In this review, a redox set-point is operationally defined not by absolute ROS levels, but by the capacity of a cellular system to sustain redox-sensitive signaling within a buffered range while avoiding irreversible macromolecular damage. Accordingly, redox set-points are inferred from integrated functional readouts of ROS flux, antioxidant buffering capacity, and redox-responsive transcriptional activity rather than from static oxidant measurements alone.

### 2.2. Metabolic Diseases: Adaptive Redox Remodeling Under Chronic Nutrient Stress

In metabolic disorders such as obesity and type 2 diabetes mellitus (T2DM), chronic nutrient excess and insulin resistance impose continuous oxidative pressure on metabolic tissues. However, disease progression is not simply the result of ROS accumulation but rather reflects adaptive rewiring of redox homeostasis that supports altered metabolic states [14,15]. Mitochondrial ROS production, antioxidant enzyme induction, and redox-sensitive metabolic signaling become tightly coordinated to sustain insulin-resistant phenotypes and low-grade inflammation. Importantly, this adaptive redox remodeling promotes disease persistence by reinforcing metabolic inflexibility. Rather than restoring redox balance, compensatory antioxidant responses often stabilize dysfunctional signaling networks, thereby perpetuating insulin resistance and inflammatory stress [16,17].

### 2.3. Neurodegenerative Diseases

In contrast to metabolic diseases and cancer, neurodegenerative disorders are characterized by a gradual loss of redox adaptability. Neurons exhibit limited capacity for redox remodeling due to high metabolic demand, low antioxidant reserve, and restricted regenerative potential [18,19]. Unlike proliferative tissue, post-mitotic neurons depend on finely regulated redox signaling to maintain synaptic transmission, axonal transport, and mitochondrial integrity, rendering them intrinsically vulnerable to cumulative redox perturbations [20,21]. As a result, sustained oxidative pressure leads to cumulative damage to proteins, lipids, and nucleic acids, ultimately overwhelming redox control systems. Studies in Alzheimer’s and Parkinson’s diseases demonstrate that early-stage redox signaling alterations precede overt neurodegeneration, suggesting that disruption of redox homeostasis is an initiating event rather than a secondary consequence [22,23]. Accumulating evidence indicates that early mitochondrial redox dysfunction—characterized by impaired electron transport chain efficiency, altered NAD^+^/NADH redox balance, and defective CoQ-dependent redox cycling—manifests initially as synaptic dysfunction and bioenergetic failure prior to irreversible neuronal loss [24,25]. These early redox disturbances compromise neuronal metabolic flexibility and calcium homeostasis, progressively eroding cellular resilience. As disease progresses, failure to maintain controlled redox signaling transitions into irreversible oxidative stress, driving neuronal dysfunction and cell loss. At advanced stages, collapse of neural redox plasticity is further exacerbated by glial dysfunction and chronic neuroinflammatory signaling, which amplify ROS and constrain compensatory antioxidant responses [26,27]. Together, these observations indicate that neurodegeneration represents a form of chronic disease adaptation in which progressive loss of redox plasticity transforms regulated redox signaling into irreversible oxidative injury.

### 2.4. Cardiovascular and Inflammatory Diseases

In chronic inflammatory and cardiovascular diseases, redox signaling acts as a central integrator of immune activation, tissue remodeling, and vascular dysfunction. Persistent ROS generation from immune cells and stressed tissues sustains NF-κB- and HIF-dependent transcriptional programs that reinforce inflammation and fibrosis [28]. Crucially, these redox-dependent signaling cascades do not resolve following the initial insult but instead become stabilized through feedforward loops involving inflammatory mediators, hypoxia signaling, and mitochondrial dysfunction. Clinical and translational studies increasingly demonstrate that chronic cardiovascular and inflammatory diseases are characterized not by transient oxidative injury but by persistent redox signaling states that actively maintain pathological tissue phenotypes. For example, low-grade inflammation in cardiometabolic disorders is associated with sustained redox-sensitive activation of innate immune pathways and endothelial dysfunction, which together drive progressive vascular remodeling rather than acute cell death [29,30]. In heart failure and atherosclerotic disease, redox-regulated inflammatory markers such as IL-6, TNF-α, and soluble urokinase plasminogen activator receptor (suPAR) correlate with disease severity and adverse clinical outcomes, underscoring the clinical relevance of maladaptive redox signaling [29,31]. These disease states exemplify redox entrenchment, in which inflammatory and metabolic signals mutually reinforce ROS-dependent transcriptional programs, thereby rendering tissues resistant to resolution despite ongoing injury. This framework explains why antioxidant therapies have largely failed in chronic inflammatory and cardiovascular diseases: suppressing ROS globally does not dismantle the underlying redox signaling architecture that sustains pathological tissue states [29,30]. Indeed, indiscriminate antioxidant intervention may inadvertently stabilize compensatory redox circuits, thereby preserving inflammatory memory and maladaptive vascular responses [32,33]. Instead, accumulating evidence supports the necessity of targeted modulation of redox-sensitive signaling nodes—such as NF-κB, HIF, and mitochondrial redox regulators—to disrupt chronic inflammatory trajectories and restore tissue adaptability. Therapeutic strategies that selectively recalibrate redox signaling, rather than abolishing ROS entirely, show greater promise in modifying disease progression across cardiovascular and inflammatory disorders [33,34]. Thus, chronic metabolic and inflammatory diseases exemplify maladaptive disease adaptation in which redox signaling becomes stabilized to perpetuate tissue remodeling and immune activation rather than restore physiological homeostasis.

### 2.5. Redox Homeostasis Breakdown as a Unifying Principle of Chronic Disease

Collectively, these findings support a unifying concept: chronic diseases arise not from excessive oxidative damage alone, but from breakdown and maladaptive reconfiguration of redox homeostasis. While the specific manifestations differ across disease contexts, the common denominator is persistent redox signaling that stabilizes pathological cellular identities and limits adaptive capacity [35,36]. Recognizing chronic diseases as disorders of redox homeostasis provides a conceptual framework for understanding why conventional antioxidant strategies have failed and why context-dependent redox modulation may offer more effective therapeutic avenues.

## 3. Redox Signaling Nodes Governing Chronic Disease Adaptation

### 3.1. NRF2 as a Master Regulator of Redox Adaptation

NRF2 (Nuclear factor erythroid 2–related factor 2) is widely recognized as the central transcriptional regulator of cellular redox homeostasis, orchestrating antioxidant defense, metabolic rewiring, and stress tolerance across chronic diseases. Under physiological conditions, NRF2 activity is tightly restrained by Kelch-like ECH-associated protein 1 (KEAP1), which functions as a redox-sensitive cysteine-based sensor that targets NRF2 for proteasomal degradation. Transient oxidative cues disrupt KEAP1–NRF2 interaction, allowing controlled nuclear translocation of NRF2 and induction of cytoprotective genes. In chronic disease states—including cancer, metabolic disorders, and neurodegeneration—NRF2 signaling frequently shifts from adaptive to constitutive activation. Sustained NRF2 activity drives persistent upregulation of glutathione synthesis (GCLC, GCLM), NADPH-generating enzymes, thioredoxin systems, and drug efflux transporters, thereby establishing a redox-adapted state rather than resolving oxidative imbalance. Importantly, this adaptive buffering enables cells to tolerate elevated ROS without triggering apoptosis or ferroptosis, fundamentally altering therapeutic vulnerability [37,38]. Recent studies further indicate that NRF2 activation extends beyond antioxidant defense to actively reprogram cellular metabolism, including enhancement of the pentose phosphate pathway, serine–glycine metabolism, and mitochondrial NADPH production, thereby coupling redox buffering with anabolic and survival pathways [39,40]. This metabolic–redox coupling allows diseased cells to maintain signaling-competent ROS levels while avoiding oxidative catastrophe, reinforcing disease persistence under chronic stress conditions [40]. In cancer, NRF2 hyperactivation is increasingly recognized as a driver of therapy resistance not only by detoxifying ROS, but by reshaping drug metabolism, ferroptosis sensitivity, and immune interactions within the tumor microenvironment [41,42]. NRF2-driven expression of SLC7A11, GPX4, and NADPH-generating enzymes establishes a ferroptosis-resistant phenotype, effectively narrowing the therapeutic window for redox-based interventions [41]. Conversely, in neurodegenerative diseases, age- and disease-associated decline of NRF2 signaling contributes to impaired mitochondrial quality control, defective antioxidant induction, and progressive neuronal vulnerability, underscoring a context-dependent duality of NRF2 function across chronic diseases [40,42]. Recent redox biology frameworks emphasize that NRF2-driven “redox homeodynamics” rather than static homestasis, under chronic disease persistence, as redox signaling becomes dynamically tuned to environmental and metabolic stressors [43,44]. Accordingly, persistent NRF2 activation should be viewed not as a compensatory antioxidant response, but as a central driver of chronic disease adaptation that stabilizes pathological redox states across cancer and related disorders.

### 3.2. FOXO Transcription Factors: Redox Sensors Linking Stress Resistance and Cell Fate

FOXO (Forkhead box O) transcription factors represent another critical redox-sensitive node that integrates oxidative signals with decisions governing cell cycle arrest, apoptosis, autophagy, and longevity. FOXO proteins are regulated through redox-dependent post-translational modifications and growth factor signaling, particularly via PI3K–AKT–mediated phosphorylation that promotes cytoplasmic sequestration. Under moderate oxidative conditions, FOXO activation induces antioxidant enzymes such as manganese superoxide dismutase (SOD2) and catalase, reinforcing redox buffering and promoting cellular survival. However, chronic activation of FOXO signaling in disease contexts often reflects an adaptive stress-resistance program rather than tumor-suppressive activity. In metabolically stressed or therapy-exposed cells, FOXO-driven transcription contributes to quiescence, dormancy, and resistance to cytotoxic insults [45]. At the molecular level, FOXO factors function as redox sensors through reversible cysteine modifications and redox-sensitive post-translational regulation, allowing FOXO activity to scale dynamically with intracellular ROS levels rather than acting as a binary on–off switch [46,47]. This property enables FOXO to orchestrate graded stress-response programs that balance antioxidant defense, metabolic restraint, and survival under persistent oxidative pressure [47]. FOXO signaling is tightly coupled to insulin//IGF–PI3K–AKT pathways, positioning FOXO as a critical interface between endocrine signaling, metabolic status, and redox control [48]. In conditions of insulin resistance or growth factor dysregulation, sustained FOXO activation promotes cell cycle arrest and metabolic conservation, phenotypes that favor disease persistence rather than elimination [48,49]. Emerging evidence further indicates that FOXO cooperates with other redox-regulated transcriptional programs, including NRF2 and xenobiotic-responsive nuclear receptors, to stabilize redox-adapted cellular states [46,50]. This cooperative network supports antioxidant capacity, autophagy, and mitochondrial quality control, collectively increasing tolerance to oxidative and therapeutic stress [50,51]. In non-malignant chronic diseases, FOXO activation plays a protective role by preserving tissue integrity under oxidative stress, as demonstrated in cardiomyocytes and neuronal systems. Loss of FOXO activity in these contexts exacerbates oxidative damage, mitochondrial dysfunction, and cell death, underscoring the context-dependent role of FOXO signaling in redox biology [51,52]. Emerging evidence indicates that FOXO signaling cooperates with NRF2 to stabilize redox-adapted phenotypes, particularly under endocrine and metabolic stress, blurring the classical distinction between tumor-suppressive and pro-survival functions [15,53]. In this context, FOXO signaling contributes to chronic disease adaptation by reinforcing stress tolerance and cellular persistence rather than terminal cell fate decisions.

### 3.3. HIF Signaling: Redox–Hypoxia Coupling in Chronic Adaptation

Hypoxia-inducible factors (HIFs) function as redox-sensitive transcriptional regulators that link oxygen availability, mitochondrial metabolism, and ROS signaling. Although traditionally viewed as hypoxia sensors, HIFs are now understood to respond to redox fluctuations independent of oxygen tension, particularly through mitochondrial ROS and redox-modulated prolyl hydroxylase activity [54]. Experimental and clinical evidence indicated that mitochondrial electron transport-derived ROS act as upstream modulators of HIF stabilization even under normoxic conditions, thereby uncoupling HIF signaling from absolute oxygen availability [55,56]. This redox-dependent regulation allows HIFs to function as integrators of metabolic stress, nutrient availability, and mitochondrial redox state rather than as passive oxygen gauges. In chronic diseases, sustained HIF activation drives angiogenesis, metabolic rewiring toward glycolysis, immune suppression, and resistance to oxidative injury. Notably, prolonged HIF signaling promotes a shift away from oxidative phosphorylation that limits excessive mitochondrial ROS generation while preserving redox signaling competence, therapy supporting long-term cellular survival under stress [57,58]. This metabolic adaptation reduces oxidative liability without restoring physiological redox balance. Importantly, HIF signaling frequently intersects with NRF2 and FOXO pathways, forming a cooperative network that promotes redox tolerance rather than hypoxic stress resolution [18,10]. Recent system-level analysis demonstrates that HIF-NRF2-FOXO crosstalk operates as a coordinated redox-adaptive module, in which transient activation supports stress resistance, whereas chronic co-activation stabilizes maladaptive disease states [59,60]. This convergence enables disease cells to repurpose ROS as signaling intermediates instead of lethal agents. Such redox–hypoxia coupling explains why interventions aimed solely at reducing oxidative stress or improving oxygenation often fail to disrupt disease progression: the dominant signaling output remains adaptive rather than cytotoxic. Indeed, therapeutic strategies that blunt ROS globally or normalize oxygen delivery fail to dismantle HIF-centered redox signaling circuits that have already been epigenetically and metabolically reinforced during chronic disease progression [55,61]. This redox–hypoxia coupling exemplifies a broader adaptive strategy in chronic disease, whereby ROS are repurposed as signaling intermediates to sustain pathological cellular states.

### 3.4. NF-κB as a Redox-Inflammatory Integrator

NF-κB occupies a unique position at the intersection of redox signaling, inflammation, and immune regulation. Unlike NRF2 and FOXO, which primarily regulate intracellular adaptation, NF-κB governs intercellular communication by controlling cytokine production, immune cell recruitment, and stromal remodeling. NF-κB activation is highly sensitive to redox cues, with both excessive ROS and reductive stress capable of modulating IκB degradation and transcriptional activity. In chronic inflammatory diseases, persistent NF-κB signaling reinforces a cytokine-rich microenvironment that stabilizes redox-adapted states rather than resolving oxidative imbalance [19,62]. Recent evidence demonstrates that ROS do not merely activate NF-κB as a by-product of inflammation, but function as upstream modulators that fine-tune NF-κB signaling amplitude, duration, and transcriptional selectivity through redox-sensitive cysteine residues within IKK and upstream adaptor complexes [63,64]. This redox-dependent tuning allows NF-κB to transition from an acute defense mediator into a chronic inflammatory driver, sustaining pathological tissue states even in the absence of ongoing injury [65]. Crucially, NF-κB and NRF2 signaling often exhibit antagonistic yet context-dependent interactions. While acute NRF2 activation suppresses inflammatory signaling, chronic disease states frequently display paradoxical NRF2–NF-κB co-activation, particularly under metabolic or endocrine stress, enabling simultaneous antioxidant buffering and inflammatory persistence [66]. Such co-activation supports immune evasion, stromal activation, and therapeutic resistance, especially in advanced cancer and cardiometabolic disease contexts. Clinical and experimental studies further reveal that global antioxidant interventions fail to dismantle NF-κB-centered inflammatory circuits because ROS suppression does not interrupt redox-dependent transcriptional memory embedded within NF-κB signaling networks [66,67]. In some settings, excessive antioxidant buffering may even prolong NF-κB-mediated cytokine signaling by preventing ROS-dependent negative feedback mechanisms, providing a mechanistic explanation for the “antioxidant paradox” observed in chronic inflammatory disease trials [68]. Thus, NF-κB functions as a redox–inflammatory integrator that reinforces chronic disease adaptation by stabilizing cytokine-driven redox remodeling rather than resolving oxidative imbalance.

### 3.5. Network-Level Integration of Redox Nodes

Rather than functioning as isolated pathways, NRF2, FOXO, HIF, and NF-κB form an interconnected redox signaling network that governs disease adaptation. The balance and temporal dynamics among these nodes determine whether ROS act as signaling messengers or cytotoxic agents. In chronic diseases, this network is progressively rewired toward tolerance, buffering, and survival. This network-level perspective provides a conceptual framework for understanding how identical redox perturbations can produce divergent biological outcomes across different disease states and systemic contexts. Their efficacy is dictated not by intrinsic chemical properties alone but by the pre-existing configuration of redox signaling nodes within the target tissue. Taken together, these network-level interactions reveal how chronic disease adaptation emerges through coordinated remodeling of redox signaling pathways rather than isolated pathway dysfunction.

## 4. Cancer as a Model of Redox Homeostatic Failure

### 4.1. Redox Homeostasis in Cancer

Cancer has long been associated with elevated oxidative stress, reflected by increased levels of ROS, oxidative DNA damage, and lipid peroxidation. Early models framed tumorigenesis as a consequence of excessive ROS-induced genomic instability. However, accumulating evidence indicates that cancer cells do not simply suffer from oxidative stress but instead operate within a reprogrammed redox homeostatic regime that actively supports malignant progression [69,70]. Recent integrative studies demonstrate that oncogenic transformation is accompanied by a coordinated rewiring of mitochondrial redox metabolism, metal ion homeostasis, and antioxidant systems, enabling sustained ROS signaling without triggering catastrophic oxidative damage [71,72]. Rather than representing a failure of antioxidant defense, cancer-associated redox imbalance reflects a shift from physiological redox signaling toward a pathologically stabilized redox state. In this state, ROS production is persistently elevated yet tightly buffered, allowing cancer cells to exploit redox signaling for proliferation, survival, and metabolic adaptation without triggering cell death [73,74]. This buffering is achieved through upregulation of glutathione, thioredoxin, and NADPH-generating pathways, as well as selective modulation of mitochondrial function and redox-active micronutrients such as copper and iron, which act as signaling cofactors rather than purely toxic species [72,75]. These adaptations permit cancer cells to maintain ROS within a signaling-competent range while suppressing lipid peroxidation–driven cell death pathways. Importantly, emerging evidence links this pathological redox control to immune evasion and therapy resistance, as stabilized redox states support PD-L1 expression, cytokine remodeling, and resistance to ferroptosis and immunogenic cell death [76,77]. Thus, cancer-associated redox imbalance should not be interpreted as uncontrolled oxidative injury, but as an actively maintained adaptive state. This distinction positions cancer not merely as a disease of oxidative damage, but as a disorder of redox homeostatic control. Viewing cancer through this lens reframes ROS from passive by-products of malignancy to functional signaling intermediates whose spatial, temporal, and quantitative regulation determines tumor fitness, therapeutic vulnerability, and disease progression [78]. Collectively, these features position cancer as a prototypical chronic disease in which redox homeostasis is not disrupted by failure, but actively remodeled to support malignant adaptation.

### 4.2. NRF2 Signaling Dysregulation and Redox Addiction in Cancer

Among redox-regulatory pathways, NRF2 signaling plays a central role in shaping cancer-associated redox phenotypes. While transient NRF2 activation protects normal cells from oxidative injury, many cancers exhibit constitutive or dysregulated NRF2 activity driven by KEAP1 mutations, oncogenic signaling, or metabolic stress [69,79]. Sustained NRF2 activation induces antioxidant enzymes, glutathione biosynthesis, NADPH regeneration, and drug efflux transporters, collectively establishing a state of redox addiction. In this context, cancer cells become dependent on NRF2-mediated buffering to survive their intrinsically high ROS burden. This redox addiction enables tolerance to oncogene-induced oxidative stress, chemotherapeutic insult, and inflammatory cues, while simultaneously narrowing the window for ROS-mediated cytotoxicity [80,81]. Importantly, this addiction does not eliminate ROS but recalibrates redox thresholds, allowing ROS to function as signaling intermediates rather than lethal agents.

### 4.3. Metabolic Rewiring, ROS Production, and Redox Dependency

Cancer-associated metabolic rewiring is inseparable from redox homeostatic failure. Enhanced glycolysis, altered mitochondrial respiration, and increased flux through anabolic pathways collectively reshape intracellular ROS generation. Mitochondria, in particular, serve as both major sources and targets of ROS, linking metabolic plasticity to redox signaling dynamics [70,82]. Rather than minimizing ROS output, cancer cells strategically balance mitochondrial ROS production with reinforced antioxidant systems. This balance supports redox-sensitive signaling pathways involved in proliferation, stemness, and therapy resistance [74,83]. Consequently, metabolic rewiring in cancer should be viewed not simply as an energy adaptation but as a redox-optimized metabolic architecture that sustains malignant phenotypes under chronic stress.

### 4.4. Mitochondrial Redox Imbalance as a Driver of Malignant Plasticity

Mitochondrial redox imbalance represents a defining feature of cancer-associated redox homeostatic failure. Altered electron transport chain activity, mitochondrial DNA mutations, and dysregulated mitochondrial dynamics contribute to sustained ROS emission that fuels oncogenic signaling [82,84]. Importantly, cancer mitochondria are not uniformly dysfunctional; instead, they exhibit selective redox dysregulation that preserves ATP production while amplifying redox signaling outputs. This mitochondrial redox imbalance enhances cellular plasticity by promoting adaptive responses to hypoxia, nutrient limitation, and therapeutic stress. Through redox-sensitive transcriptional programs, cancer cells exploit mitochondrial ROS to coordinate metabolic flexibility, epithelial–mesenchymal transition, and survival under hostile microenvironmental conditions [85,86].

### 4.5. Redox Signaling Plasticity and Therapeutic Implications

A defining characteristic of cancer as a redox disease is its pronounced redox signaling plasticity. Cancer cells dynamically adjust redox signaling intensity, spatial distribution, and downstream responses in accordance with microenvironmental pressures and treatment exposure [84,87]. This plasticity allows tumors to evade oxidative collapse while maintaining responsiveness to redox cues that support growth and dissemination. From this perspective, cancer represents a paradigmatic model of redox homeostatic failure—not because redox control is lost, but because it is pathologically repurposed. Therapeutic strategies that indiscriminately suppress ROS or enhance antioxidant capacity may therefore stabilize redox-adapted tumor states rather than disrupt them. Effective redox-targeted interventions must instead account for the underlying redox dependency and signaling plasticity that define cancer progression.

### 4.6. Is Cancer a Redox Disease?

Collectively, these observations support the view that cancer can indeed be considered a redox disease, characterized by maladaptive stabilization of redox homeostasis rather than uncontrolled oxidative stress. This perspective provides insight into redox-modulating therapies that often produce context-dependent outcomes and highlights the necessity of precision redox intervention strategies guided by disease state, metabolic state, and redox biomarkers.

## 5. Therapeutic Redox Reprogramming in Cancer

Accumulating evidence indicates that effective redox-based cancer therapy does not rely on indiscriminate suppression or amplification of ROS, but rather on strategic modulation of tumor redox homeostasis toward states that compromise malignant fitness [88,89]. Cancer cells operate within a narrowly defined redox window—often referred to as a redox set-point—that supports oncogenic signaling, metabolic flexibility, and therapeutic tolerance [90,91]. Therapeutic success therefore depends on disrupting this optimized redox equilibrium.

### 5.1. Redox Set-Point and Tumor Vulnerability

In this section, redox addiction refers to pathological dependence on a narrowly buffered ROS signaling window that maintains adaptive signaling without triggering oxidative damage. Cancer cells maintain elevated basal ROS levels relative to normal tissues, yet simultaneously reinforce antioxidant systems to prevent lethal oxidative damage [89,90]. This paradoxical state reflects a redox addiction, whereby tumor growth and survival depend on sustained but tightly buffered ROS signaling. Key redox-buffering mechanisms—including NRF2-driven antioxidant transcription, glutathione and thioredoxin systems, NADPH-generating metabolic pathways, and mitochondrial adaptations—collectively define the tumor-specific redox set-point [90,92]. Therapeutic redox reprogramming aims to shift this set-point beyond the adaptive range of cancer cells. Importantly, this can be achieved through bidirectional strategies: either by overwhelming antioxidant capacity to induce oxidative collapse, or by suppressing adaptive redox signaling nodes required for stress tolerance and survival [91,92,93]. The feasibility of each approach is highly context-dependent and varies across tumor type, metabolic state, and treatment history.

### 5.2. Restoring vs. Disrupting Redox Balance: A False Dichotomy

Early antioxidant-based clinical strategies were predicated on the assumption that restoring “normal” redox balance would suppress tumor progression. However, large-scale failures of non-specific antioxidant supplementation highlighted a fundamental misconception: cancer redox homeostasis is not simply elevated oxidative stress, but an actively remodeled signaling architecture [88,89]. In many contexts, antioxidant interventions may inadvertently stabilize tumor redox buffering and reinforce therapy resistance. By contrast, contemporary redox-targeted strategies emphasize selective disruption of redox control circuits rather than normalization. Targeting metabolic–redox nodes such as serine/glycine one-carbon metabolism, glutamine utilization, cystine uptake (xCT), or mitochondrial NADPH production can selectively impair the reducing capacity of cancer cells while sparing normal tissues [92,94,95]. These interventions expose a therapeutic vulnerability intrinsic to redox-adapted tumors.

### 5.3. Combination Redox Modulation as a Therapeutic Principle

A recurring theme across experimental and translational studies is that monotherapy targeting redox pathways is rarely durable, owing to rapid compensatory rewiring [90,93]. Cancer cells exhibit remarkable plasticity, rerouting metabolic fluxes and antioxidant defenses to re-establish redox equilibrium under therapeutic pressure. Consequently, redox reprogramming is most effective when implemented as part of rational combination strategies. Combining redox-modulating agents with chemotherapy, targeted therapy, radiotherapy, or immunotherapy can exploit therapy-induced oxidative stress and prevent adaptive buffering [90,95]. For example, inhibition of antioxidant defenses can sensitize tumors to DNA-damaging agents, while metabolic redox disruption can impair stemness maintenance and overcome drug resistance [91,96]. Importantly, these strategies do not rely on maximal ROS induction but on timed and spatially controlled redox imbalance.

### 5.4. Context-Dependent Redox Therapy and Precision Stratification

The therapeutic outcome of redox reprogramming is strongly shaped by tumor context, including metabolic state, hypoxic burden, mitochondrial function, and prior treatment exposure [89,92]. Tumors characterized by high NRF2 activity, enhanced glutathione synthesis, or stem-like phenotypes may require distinct redox-targeting strategies compared to redox-fragile or metabolically constrained cancers. Emerging evidence supports the use of redox biomarkers—such as NRF2 pathway activation, xCT/GPX4 expression, NADPH/GSH ratios, or metabolic enzyme dependencies—to guide patient stratification and therapeutic design [93,96]. Such precision-oriented approaches transform redox modulation from a generalized concept into a context-aware therapeutic framework.

### 5.5. Conceptual Shift: From Oxidative Stress to Redox Reprogramming

**Figure 1:**
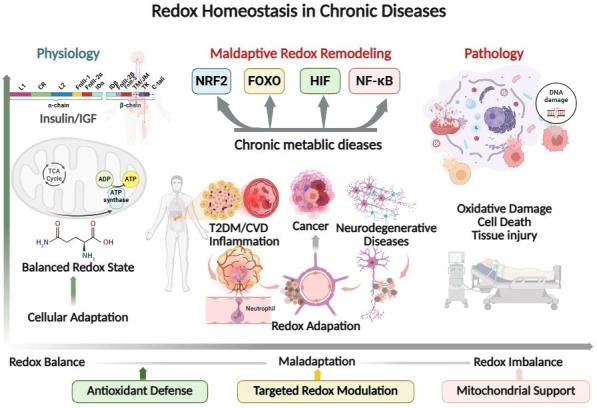
Redox homeostasis as a dynamic continuum in chronic diseases. Physiological redox signaling supports cellular adaptation and metabolic homeostasis under tightly regulated reactive oxygen species (ROS) levels. Chronic endocrine, metabolic, and inflammatory stressors progressively drive maladaptive redox remodeling through key regulatory nodes, including NRF2, FOXO, HIF, and NF-κB. This adaptive shift underlies diverse chronic disease states, such as cardiometabolic disorders, cancer, and neurodegenerative diseases, which exhibit distinct redox set-points and therapeutic vulnerabilities. Effective intervention therefore requires context-dependent redox modulation rather than indiscriminate antioxidant supplementation. Abbreviations: Cardiovascular disease (CVD) Forkhead box O (FOXO), Hypoxia-inducible factor (HIF), Insulin-like growth factor (IGF), Nuclear factor erythroid 2–related factor 2 (NRF2), Nuclear factor kappa B (NF-κB), Reactive oxygen species (ROS), Type 2 diabetes mellitus (T2DM).

Collectively, these findings necessitate a conceptual shift in redox-based cancer therapy. Rather than viewing ROS as uniformly harmful entities to be suppressed, redox signaling must be understood as a dynamic, rewired system that supports malignancy. Therapeutic redox reprogramming seeks to dismantle this system by selectively targeting its regulatory nodes, metabolic supports, and adaptive thresholds [88,90]. As summarized in Figure 1, chronic diseases are not defined by a linear progression from redox balance to oxidative damage, but rather by intermediate states of maladaptive redox remodeling, in which transcriptional regulators such as NRF2, FOXO, HIF, and NF-κB stabilize disease-specific redox adaptations. Cancer represents a paradigmatic example of this process, while cardiometabolic and neurodegenerative diseases exhibit structurally analogous redox remodeling trajectories.

## 6. Natural Compounds as Redox Modulators

Natural compounds remain highly relevant in redox-based cancer therapy not because they function as simple antioxidants, but because they act as context-sensitive modulators of redox signaling architecture rather than indiscriminate scavengers of ROS [97]. Accumulating evidence demonstrates that many phytochemicals possess intrinsic redox activity that allows them to reshape cellular redox set-points, influence mitochondrial signaling, and recalibrate stress-adaptive pathways that are dysregulated in cancer [98,99]. Unlike conventional antioxidants designed to globally suppress oxidative stress, natural compounds frequently exhibit biphasic or hormetic redox behavior, functioning as antioxidants under conditions of excessive oxidative burden while exerting pro-oxidant or signaling-modulatory effects within redox-adapted tumor cells [100]. This duality enables phytochemicals to interact dynamically with endogenous redox networks rather than overriding them, a property that is increasingly recognized as essential for therapeutic efficacy in chronic redox-remodeled diseases such as cancer [101].

At the molecular level, phytochemicals modulate redox signaling through multiple mechanisms, including regulation of cysteine-based redox switches, modulation of antioxidant response elements, and interference with redox-sensitive transcription factors such as NRF2, NF-κB, and HIF-1α [97,98]. Importantly, these effects are rarely confined to a single pathway. Instead, natural compounds often exert network-level redox modulation, simultaneously influencing metabolic flux, inflammatory signaling, and epigenetic regulation [102,103]. Mitochondria represent a central hub through which many natural compounds exert their redox-modulatory effects. Numerous phytochemicals directly or indirectly influence mitochondrial electron transport chain activity, mitochondrial ROS generation, and redox-coupled apoptotic signaling [103,104]. In this context, emerging multi-component phytochemical formulations designed to target redox signaling networks—rather than single oxidative endpoints—illustrate the translational feasibility of redox-guided cancer therapy. Such formulations integrate mitochondrial redox modulation, attenuation of adaptive antioxidant buffering, and interference with redox-dependent survival signaling, thereby aligning closely with the principles of redox reprogramming described above [104,105]. Representative examples include multi-herbal formulations shown to modulate redox buffering capacity and redox-sensitive signaling programs, with selected formulations suggesting direct or indirect regulation of NRF2-centered pathways and broader functional engagement of FOXO-, HIF-, and NF-κB-associated redox processes in preclinical cancer models (e.g., BK002-, SH003-, JI017-, and aRVS-based formulations).

Another defining feature of natural compounds is their ability to interact synergistically with existing therapies through redox modulation. Rather than acting as standalone cytotoxic agents, phytochemicals frequently enhance the efficacy of chemotherapy, radiotherapy, or targeted agents by shifting redox thresholds, suppressing adaptive antioxidant responses, or attenuating redox-driven drug resistance mechanisms [98,106]. This combination potential is particularly relevant in tumors characterized by high redox plasticity, where single-agent redox perturbation is readily neutralized by compensatory buffering systems. Importantly, the therapeutic relevance of natural compounds must be interpreted within a context-dependent framework. Their efficacy is strongly influenced by tumor redox state, metabolic configuration, mitochondrial function, and microenvironmental conditions, including inflammation and nutrient availability [99]. Failure to account for these contextual determinants has contributed to inconsistent outcomes in both preclinical and clinical studies, underscoring the need for biomarker-guided application of phytochemical-based redox therapies. Beyond their intrinsic redox-modulatory properties, the biological activity of natural compounds is profoundly shaped by systemic hormonal states that precondition cellular redox responsiveness. Emerging evidence indicates that endocrine signals such as insulin/IGF-1, glucocorticoids, and sex hormones actively define cellular hormetic windows by regulating mitochondrial activity, antioxidant capacity, and redox-sensitive transcriptional programs [106,107]. Within this hormone-conditioned redox landscape, phytochemicals do not act as isolated biochemical agents but rather as modulators whose efficacy depends on pre-existing endocrine and metabolic cues.

Insulin and IGF-1 signaling, in particular, have been shown to suppress FOXO-dependent antioxidant programs while enhancing PI3K–AKT–mTOR–driven metabolic flux, thereby shifting redox set-points toward adaptive tolerance rather than vulnerability [106,107]. Under such conditions, natural compounds that rely on redox perturbation to induce cytotoxic stress may exhibit attenuated efficacy, as hormone-driven buffering systems absorb redox fluctuations without triggering irreversible damage. Conversely, phytochemicals capable of interfering with hormone-linked redox signaling—rather than simply increasing ROS—may selectively destabilize these adaptive states. Stress-associated hormones further refine the redox context in which natural compounds operate. Glucocorticoid signaling has been shown to reprogram mitochondrial redox balance and antioxidant gene expression, influencing cellular susceptibility to redox-active interventions [107]. Similarly, circadian and neuroendocrine regulators such as melatonin modulate mitochondrial ROS production and redox signaling amplitude, thereby altering the temporal and contextual responsiveness of cells to phytochemical exposure [108]. These findings suggest that the same natural compound may exert divergent redox effects depending on endocrine timing, hormonal tone, and metabolic state.

Taken together, these observations reinforce the concept that natural compounds function most effectively as redox modulators when considered within a hormone-conditioned framework. Their therapeutic relevance arises not from universal antioxidant capacity but from their ability to interact with endocrine-regulated redox networks that govern cellular adaptation, stress tolerance, and fate decisions. Failure to account for hormonal context may therefore obscure the true mechanism of action of phytochemicals and contribute to inconsistent experimental and clinical outcomes [106,107,108]. In this broader context, natural compounds remain viable and scientifically grounded therapeutic agents not because they universally suppress oxidative stress, but because they function as adaptive redox modulators capable of reprogramming dysregulated redox signaling networks. When applied with mechanistic insight and contextual precision, phytochemicals offer a unique opportunity to exploit redox vulnerabilities in cancer while minimizing collateral disruption of physiological redox signaling. This conceptual shift—from antioxidant supplementation to redox reprogramming—provides a rational framework for integrating natural compounds into modern redox-targeted cancer therapy [97,102,103].

## 7. Clinical Implications and Future Directions

The translational failure of many redox-targeted interventions has underscored a central limitation in current clinical paradigms: redox imbalance is still treated as a uniform biochemical abnormality rather than a context-dependent regulatory state. Accumulating evidence from oncology, metabolic disease, and neurodegeneration indicates that therapeutic success depends less on global suppression of ROS and more on precise alignment between disease stage, redox set-point, and adaptive capacity of affected tissues [108,109]. Accordingly, future redox medicine must transition from nonspecific antioxidant supplementation toward patient-stratified, mechanism-informed redox modulation.

### 7.1. Patient Stratification Based on Redox States

One of the most critical unmet needs in clinical redox medicine is the lack of stratification frameworks that distinguish oxidative distress from adaptive redox signaling. Tumors and chronic disease tissues frequently exist in redox-adapted states characterized by elevated but buffered ROS, reinforced antioxidant programs, and rewired stress-response pathways. In such settings, indiscriminate antioxidant therapy may stabilize disease rather than disrupt it [110]. Clinical stratification should therefore incorporate functional redox profiling—distinguishing redox-vulnerable from redox-buffered states—prior to therapeutic intervention.

### 7.2. Redox Biomarkers as Decision-Making Tools

Progress in translational redox medicine will depend on the integration of robust, clinically actionable biomarkers. Emerging studies highlight the value of combining systemic markers (e.g., circulating 4-hydroxynonenal, F2-isoprostanes, oxidized glutathione ratios) with pathway-specific indicators such as NRF2 target gene expression, thioredoxin system activity, and mitochondrial redox signatures [111,112]. Importantly, biomarker panels should reflect dynamic redox regulation rather than static oxidative damage, enabling real-time assessment of therapeutic response and redox reprogramming efficacy. The clinical implications of redox dysregulation differ substantially across chronic diseases depending on the underlying redox set-point and adaptive capacity. As summarized in Table 1, cancer, cardiometabolic disorders, and neurodegenerative diseases exhibit distinct redox homeostatic architectures that necessitate fundamentally different therapeutic strategies, ranging from redox destabilization to redox restoration.

### 7.3. Disease-Stage–Specific Redox Targeting

Redox dependency evolves during disease progression. Early-stage tumors and pre-malignant lesions often retain limited buffering capacity and remain sensitive to redox perturbation, whereas advanced cancers exhibit reinforced antioxidant defenses and metabolic plasticity. Clinical strategies must therefore adapt redox interventions to disease stage—employing redox-disruptive approaches in adaptive tumors while favoring redox-restorative or immunomodulatory strategies in inflammatory or degenerative contexts [42,112]. Failure to account for temporal redox evolution likely explains inconsistent outcomes across prior clinical trials.

### 7.4. Toward Personalized Redox Therapy

The convergence of multi-omics profiling, redox metabolomics, and computational modeling now enables individualized mapping of redox signaling networks. Precision redox medicine envisions therapeutic regimens tailored to each patient’s redox architecture, integrating antioxidant capacity, mitochondrial function, inflammatory tone, and hormonal status [42,112]. Such personalization is particularly relevant in cancer, where redox signaling intersects with immune evasion, therapy resistance, and metabolic rewiring.

### 7.5. Translational Redox Medicine: From Concept to Clinic

Ultimately, the future of redox-targeted therapy lies in abandoning the outdated dichotomy of “antioxidants versus pro-oxidants” and embracing redox reprogramming as a systems-level intervention. Combination strategies—pairing redox modulators with chemotherapy, immunotherapy, metabolic inhibitors, or endocrine modulation—offer the greatest promise for exploiting disease-specific redox vulnerabilities while preserving physiological signaling [108,121]. This paradigm shift reframes redox biology not as a background stress response, but as a programmable determinant of therapeutic outcome. To translate redox signaling concepts into clinically actionable strategies, it is essential to align disease-specific redox states with appropriate therapeutic directions. Table 2 summarizes representative redox biomarkers and corresponding intervention strategies across major chronic disease contexts. These stratification frameworks further emphasize that redox modulation must be tailored not only to disease type but also to disease stage and adaptive capacity, a principle that extends beyond oncology to cardiometabolic and neurodegenerative disorders.

### 7.6. Implications Beyond Cancer: Cardiometabolic and Neurodegenerative Diseases

In this section, cancer is treated as a paradigmatic but not exclusive model of maladaptive redox homeostasis. This framework proposed here does not assume mechanistic equivalence across chronic diseases but instead emphasizes shared regulatory principles governing redox remodeling, adaptive buffering, and signaling persistence under chronic stress. Accordingly, the extension of this framework beyond oncology is intended to highlight structural and conceptual analogies rather than disease-specific molecular identity.

While cancer provides a compelling model for redox-adaptive pathology, accumulating evidence indicates that dysregulated redox signaling constitutes a unifying pathogenic mechanism across a broad spectrum of chronic non-communicable diseases, including cardiometabolic and neurodegenerative disorders. In these contexts, disease progression is not driven by indiscriminate oxidative damage alone but rather by sustained perturbations in redox homeostasis that reshape metabolic, inflammatory, and stress-response networks. In cardiometabolic diseases, excessive nutrient flux, genetic susceptibility, and mitochondrial dysfunction converge to establish chronically altered redox states characterized by elevated ROS production coupled with maladaptive antioxidant responses. Aberrant redox signaling contributes to insulin resistance, endothelial dysfunction, and inflammatory activation, thereby promoting the progression of metabolic-associated fatty liver disease, cardiovascular disease, and chronic kidney disease [118,122]. Importantly, these conditions exhibit redox remodeling rather than simple antioxidant depletion, underscoring the limitation of nonspecific antioxidant supplementation strategies. Similarly, neurodegenerative diseases are increasingly recognized as disorders of redox signaling failure rather than isolated oxidative stress syndromes. Neurons are uniquely vulnerable to redox imbalance due to their high metabolic demand and limited regenerative capacity. Mitochondrial dysfunction, impaired redox buffering, and dysregulated antioxidant signaling pathways collectively drive progressive neuronal loss in disorders such as Alzheimer’s disease, Parkinson’s disease, and amyotrophic lateral sclerosis [116,117]. Notably, clinical and genetic evidence suggests that antioxidant interventions exert heterogeneous and context-dependent effects, reflecting the complexity of disease-specific redox architectures rather than uniform ROS toxicity. Emerging therapeutic paradigms across these chronic diseases emphasize modulation of endogenous redox signaling networks rather than global ROS suppression. Mechanism-oriented strategies targeting intrinsic antioxidant regulators—such as NRF2, DJ-1, and mitochondrial redox enzymes—have demonstrated translational relevance in cancer, cardiometabolic disorders, and neurodegeneration alike [119]. In parallel, nutritional and immunometabolic interventions that recalibrate redox–immune crosstalk are gaining attention as disease-modifying approaches in neurodegenerative conditions. Collectively, these findings support a conceptual shift in which chronic diseases are understood as states of maladaptive redox signaling rather than mere consequences of oxidative injury. This cross-disease perspective reinforces the central premise of the present review: effective therapeutic intervention requires restoration of redox homeostasis through context-aware modulation of signaling networks, a principle that transcends cancer and extends to cardiometabolic and neurodegenerative diseases. Taken together, the extension of redox signaling concepts beyond oncology underscores that context-dependent redox remodeling represents a shared pathological principle across diverse chronic diseases. Rather than being confined to malignant transformation, maladaptive redox signaling emerges as a common determinant of disease persistence, therapeutic resistance, and clinical heterogeneity in cardiometabolic and neurodegenerative disorders. These cross-disease parallels provide a unifying framework for interpreting the inconsistent outcomes of redox-targeted interventions and set the stage for a broader discussion on how redox signaling should be conceptualized and therapeutically addressed in chronic disease contexts.

## 8. Limitations

Several limitations of this review should be acknowledged. First, a substantial portion of the mechanistic and translational evidence discussed herein is derived from cancer research, where redox adaptation, therapeutic resistance, and metabolic plasticity are most extensively characterized. While cancer provides a robust and clinically relevant model for studying redox signaling dysregulation, extrapolation of these concepts to other chronic diseases must be approached with caution. Second, the redox architectures of cardiometabolic and neurodegenerative diseases exhibit tissue-specific, temporal, and cell-type–dependent features that are not fully captured by oncology-based frameworks. Although emerging data support shared principles of maladaptive redox signaling across chronic diseases, disease-specific validation—particularly in longitudinal clinical settings—remains limited. Finally, the availability of standardized and clinically validated redox biomarkers continues to constrain the translation of redox-based stratification strategies into routine practice. Future studies integrating redox metabolomics, imaging-based redox sensors, and systems-level modeling will be essential to refine patient selection and therapeutic targeting across diverse disease contexts.

## 9. Conclusions

Redox signaling constitutes a fundamental regulatory layer that shapes disease initiation, progression, and therapeutic response across a broad spectrum of chronic conditions. Accumulating evidence challenges the traditional view that oxidative stress simply reflects excessive and indiscriminate ROS accumulation, instead revealing redox imbalance as a dynamic, context-dependent signaling state. Accordingly, the clinical success or failure of redox-targeted interventions is determined not by the extent of ROS suppression, but by their alignment with disease-specific redox set-points, adaptive buffering capacity, and therapeutic timing. This review advances a translational shift toward personalized redox medicine, in which redox signaling pathways and homeostatic set-points are leveraged as actionable therapeutic targets in chronic oxidative diseases. Rather than relying on nonspecific antioxidant supplementation, effective redox intervention requires biomarker-guided stratification, disease stage-specific assessment of redox adaptability, and mechanistic understanding of pathological redox control. Within this framework, precision redox modulation emerges as a clinically relevant decision paradigm—designed to destabilize maladaptive redox adaptations while preserving physiological redox signaling. By reframing chronic diseases, particularly cancer, as disorders of maladaptive redox regulation rather than simple oxidative injury, this review provides a coherent roadmap for integrating redox biology into rational, biomarker-informed clinical decision-making.

## Figures and Tables

**Table 1:** Comparative redox homeostatic frameworks across chronic diseases and therapeutic implications.

Disease Context	Dominant Redox State	Key Signaling Features	Adaptive/Maladaptive Outcome	Therapeutic Redox Strategy	Refs
Cancer	Redox-adapted high ROS (buffered)	NRF2 activation, metabolic rewiring, mitochondrial ROS buffering	Therapy resistance, survival under stress	Redox disruption/set-point destabilization	[69,70,73,74,88]
Cardiometabolic diseases (T2DM, CVD)	Chronically shifted redox set-point	Insulin/IGF signaling, mitochondrial dysfunction, inflammatory redox signaling	Metabolic inflexibility, endothelial dysfunction	Redox recalibration/restoration of flexibility	[113,114,115,116,117]
Neurodegenerative diseases	Failed redox buffering	Mitochondrial impairment, impaired antioxidant signaling, redox–protein aggregation	Progressive neuronal loss	Redox restoration/mitochondrial support	[118,119,120]

Abbreviations: Reactive oxygen species (ROS), Nuclear factor erythroid 2–related factor 2 (NRF2), Type 2 diabetes mellitus (T2DM).

**Table 2:** Context-dependent redox biomarkers and therapeutic strategies in chronic diseases.

Clinical Context	Dominant Redox Feature	Representative Biomarkers	Therapeutic Redox Direction	Refs
Redox-adapted tumors	Buffered high ROS, elevated antioxidant capacity	NRF2-high, SLC7A11-high, GPX4-high	Redox destabilization/ferroptosis sensitization	[69,70,73,88,89]
Metabolic inflexibility (T2DM, obesity)	Chronically shifted redox set-point	Insulin-high, mitochondrial ROS, inflammatory cytokines	Redox recalibration/metabolic restoration	[113,114,115,116,117]
Neurodegenerative disorders	Failed redox buffering	Mitochondrial dysfunction markers, oxidized proteins	Redox restoration/mitochondrial support	[118,119,120]
Early-stage disease/pre-adaptive state	Preserved redox plasticity	Balanced ROS, intact antioxidant signaling	Adaptive redox modulation	[97,98,103,106,107]

Abbreviations: Glutathione Peroxidase 4 (GPX4), Nuclear factor erythroid 2–related factor 2 (NRF2), Reactive oxygen species (ROS), Solute carrier family 7 member 11 (SLC7A11), Type 2 diabetes mellitus (T2DM).

## Data Availability

No new data were created or analyzed in this study. Data sharing is not applicable to this article.
